# *Akkermansia muciniphila* in the Human Gastrointestinal Tract: When, Where, and How?

**DOI:** 10.3390/microorganisms6030075

**Published:** 2018-07-23

**Authors:** Sharon Y. Geerlings, Ioannis Kostopoulos, Willem M. de Vos, Clara Belzer

**Affiliations:** 1 Laboratory of Microbiology, Wageningen University and Research, Stippeneng 4, 6708WE Wageningen, The Netherlands; sharon.geerlings@wur.nl (S.Y.G.); ioannis.kostopoulos@wur.nl (I.K.); willem.devos@wur.nl (W.M.d.V.); 2 Immunobiology Research Program, Department of Bacteriology and Immunology, Haartman Institute, University of Helsinki, 00014 Helsinki, Finland

**Keywords:** *Akkermansia muciniphila*, mucin, gut microbiota, ecological niches, digestive tract, human breast milk

## Abstract

*Akkermansia muciniphila* is a mucin-degrading bacterium of the phylum Verrucomicrobia. Its abundance in the human intestinal tract is inversely correlated to several disease states. *A. muciniphila* resides in the mucus layer of the large intestine, where it is involved in maintaining intestinal integrity. We explore the presence of *Akkermansia*-like spp. based on its 16S rRNA sequence and metagenomic signatures in the human body so as to understand its colonization pattern in time and space. *A. muciniphila* signatures were detected in colonic samples as early as a few weeks after birth and likely could be maintained throughout life. The sites where *Akkermansia*-like sequences (including Verrucomicrobia phylum and/or *Akkermansia* spp. sequences found in the literature) were detected apart from the colon included human milk, the oral cavity, the pancreas, the biliary system, the small intestine, and the appendix. The function of *Akkermansia*-like spp. in these sites may differ from that in the mucosal layer of the colon. *A. muciniphila* present in the appendix or in human milk could play a role in the re-colonization of the colon or breast-fed infants, respectively. In conclusion, even though *A. muciniphila* is most abundantly present in the colon, the presence of *Akkermansia*-like spp. along the digestive tract indicates that this bacterium might have more functions than those currently known.

## 1. Introduction

The microbial community in the human gut plays a role in the balance between health and disease. The gastrointestinal (GI) microbiota has recently emerged as an important factor in human physiology, both under homeostatic and pathological conditions [1]. Characterization of the GI microbiota may identify gut-related abnormalities and play an important role in establishing functional linkages to health status [2]. Some GI tract disorders with associated microbiota imbalance include celiac disease [2], irritable bowel syndrome (IBS) [3,4], inflammatory bowel disease (IBD) [5,6,7], and type 2 diabetes (T2D) [8,9,10]. 

The *A. muciniphila*-type strain Muc^T^ of the phylum Verrucomicrobia was first described in 2004 [11]. This bacterium was isolated from the fecal sample of a healthy individual using purified mucin as the sole source of carbon, nitrogen, and energy for growth. *A. muciniphila* has only a few similarities to other representatives of Verrucomicrobia [12]. Interestingly, *Akkermansia* is the only genus of the Verrucomicrobia phylum found in gastrointestinal samples. Large differences were observed between verrucomicrobial genomes, in terms of the GC content and genome sizes. In contrast, similarities were observed within the Verrucomicrobia phylum as a large proportion of the proteins in verrucomicrobial proteomes were found to contain signal peptides (26.1% for *A. muciniphila*). In the colon of a healthy human being, *A. muciniphila* is present in high levels with an abundance of approximately 3% [6,13]. The core activity of *A. muciniphila* is to degrade mucus using the many mucolytic enzymes encoded in its genome [6,12].

The presence of *A. muciniphila* has been associated with healthy intestine and its abundance has been inversely correlated to several disease states [6,7,8,14,15,16]. For example, in cases of IBD (patients with ulcerative colitis and Crohn’s disease), the abundance of *A. muciniphila* was found to be decreased [6,7]. Also, individuals with acute appendicitis were found to harbor a decreased amount of *A. muciniphila* [14]. In this case, the abundance of *A. muciniphila* was inversely related to the severity of the appendicitis. Furthermore, obese children were shown to have a significant reduction in *A. muciniphila*-like bacteria [15]. In a comprehensive study of infants in daycare, *A. muciniphila*-based sequences were found to be reduced in children that had received multiple antibiotic courses and were at risk for later life obesity [17]. In concordance with these studies, another reported the association of *A. muciniphila* with a healthier metabolic status in obese and overweight individuals [16]. Moreover, the genus *Akkermansia* and its metabolic pathways were found to be enriched in athletes with a low body mass index [18,19]. Lastly, the abundance of Verrucomicrobiae was significantly reduced in pre-diabetes and T2D [8]. It is important to note that these diseases may have an effect on the integrity or thickness of the mucus layer, and thereby effect the abundance of *A. muciniphila*. To confirm this, studies taking both the microbial composition and mucus layer integrity into account should be performed. Altogether, these studies indicate the correlation of *A. muciniphila* to a healthy status and indicate the possible use of *Akkermansia* spp. as a biomarker for disease.

Interactions between the host and *A. muciniphila* have been studied in mice [10,20]. Colonization by *A. muciniphila* resulted in transcriptional changes, leading to an increase in the expression of genes associated with immune responses [20]. Furthermore, *A. muciniphila* was found to strengthen the gut barrier function in mice [10]. By doing so, *A. muciniphila* played a role in normalizing metabolic endotoxemia and adipose tissue metabolism. These findings have been supported by another study showing that *A. muciniphila* affects genes involved in cellular lipid metabolism [21]. The effect of a fiber-free diet was studied in mice colonized with a synthetic community consisting of 14 species, including *A. muciniphila* [22]. Feeding these mice a fiber-free diet was found to damage the mucus barrier. The changes in microbial community included an increased abundance of *A. muciniphila* and a switch in metabolism of gut microbiota species from fiber degradation to mucus glycan degradation [22,23]. An in vitro study using human colonic cell lines (Caco-2 and HT-29) demonstrated the adherence of *A. muciniphila* to the intestinal epithelium, thereby strengthening the epithelial integrity rather than causing a pro-inflammatory reaction [24]. Lastly, outer membrane proteins of *A. muciniphila* were found to have a role in the modulation of immune responses [25]. Recently, one of the outer membrane proteins was identified (Amuc_1100) [26]. This study demonstrated that the outer membrane pili-like protein is involved in immune regulation and the enhancement of trans-epithelial resistance.

Several studies have purposely or unintentionally revealed the presence of *Akkermansia*-like spp. in segments of the human body other than the colon, where *A. muciniphila* might also have important functions. In this review, we will discuss the presence of Verrucomicrobia and *Akkermansia*-like spp. in different anatomic regions of the digestive tract. The physiology and environmental parameters of these anatomic regions will be taken into account to assess the possibility of *A. muciniphila* to colonize and be active at these niches.

## 2. Prevalence of *Akkermansia muciniphila* through Geography and Age in the Human Gastrointestinal Tract

*Akkermansia muciniphila* is present in the intestinal tract throughout different stages of life [27,28]. This was determined using fecal samples from healthy subjects divided into groups based on their age, analyzed using fluorescence in situ hybridization (FISH) and quantitative PCR (qPCR). The number of bacteria related to *A. muciniphila* significantly increased from early life to adult subjects [27,28]. When focusing on the prevalence of bacteria related to *A. muciniphila*, 16% of one-month old infants in this study were found to harbor *Akkermansia* in their intestinal tracts [27]. At this very young age, the concentration of *A. muciniphila* ranged between 2.05 and 4.36 log cells per gram of feces. Subsequently, at 6 months of age the percentage of infants where *A. muciniphila* could be detected increased to 72%, with a further increase to 90% in 12-month-old infants. The concentrations were in the ranges of 2.50–7.30 and 2.80–9.50 log cells per gram of feces in 6-month-old and 12-month-old infants, respectively. However, in this study no correlation was made between the *A. muciniphila* abundance and the feeding mode of the infant (breast and/or formula feeding). Other studies not primarily focusing on the presence of *A. muciniphila* have also shown the presence of *Akkermansia-*like spp. or the Verrucomicrobia phylum in the infant’s gut at different geographical locations, such as Finland, Germany, and Malawi [29,30,31]. The presence of *A. muciniphila* in the infant’s gut could be considered as a marker for gut microbiota development and diversity [29]. Next to infants, adults (25–35 years old) were found to harbor 5–8.8 log cells per gram of feces of *A. muciniphila*, while a significant decrease was noted in elderly subjects (80–82 years old) namely, 95.5% [27]. These outcomes differed in another study, where young adults harbored significantly less *Akkermansia* spp. than the elderly both in terms of prevalence and abundance [32]. In addition, centenarians were found to harbor a higher concentration of *Akkermansia* spp. Also, semi-supercentenarians (individuals with an age of 105 or higher) had a higher concentration of *Akkermansia*-like spp. than other (younger) age groups in the study [33]. *A. muciniphila* is proposed to have a role in the immunological and metabolic health of semi-supercentenarians, rendering it a biomarker for healthy aging. In contrast to age, gender does not play a role considering the amount of *A. muciniphila* [28].

The genome of *A. muciniphila* (ATCC BAA-835) was sequenced and annotated [12]. These sequences were used to mine 37 reported gastrointestinal tract (GIT) metagenomes derived from adults belonging to six nationalities to evaluate the presence and genetic diversity of *Akkermansia* spp. in the human gut. The prevalence of *Akkermansia* spp. in these metagenomes was found to be 30%, using a cutoff of >95% identity to 16S rRNA. When queried with the *Akkermansia* genome (identity > 90%), 62% of the metagenomic libraries were shown to be *Akkermansia* carriers, which is comparable to earlier findings of a Finnish cohort [27]. The relative abundances of *Akkermansia* spp. DNA in these libraries varied from <0.01% to almost 4% [12]. However, higher abundances of Verrucomicrobia in the GIT have also been reported, for example in biopsy samples (up to ~15% depending on the method and individual) [34]. The analysis of fecal samples from the Metagenomics of the Human Intestinal Tract (MetaHit) project, derived from Danish and Chinese individuals, revealed country-specific differences in gut microbiota [35]. In terms of *Akkermansia*-like spp., the mean relative abundance of Danish individuals (0.0137) was higher than that of Chinese individuals (0.0015). Moreover, a recent study showed that Verrucomicrobia sequences were found to be enriched in the guts of industrialized regions compared to the guts of traditional populations (*p* < 2 × 10^−16^), such as the traditional Hadza hunter-gatherers [36]. However, as with many comparative microbiota analyses, confounding factors related to sample processing, DNA extraction, and subsequent processing cannot be ruled out [37]. A study performed in China showed that the frequency of *A. muciniphila* is lower in southern Chinese than in European populations [38]. The frequency in southern China was found to be 51.71%, which is significantly lower than the ~75% found in European populations [27,38]. Interestingly, in southern China the highest frequencies were detected among the elderly, while in European populations a significant decrease was noted for the elderly. This observed difference may be due to external factors affecting the microbiota composition, including geographic location, diet, and age.

**Figure 1:**
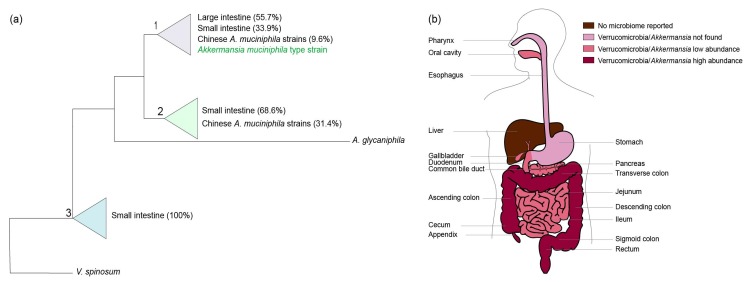
(**a**) *Akkermansia* is not only present in the large intestine, but also in other anatomic regions of the digestive tract. A schematic overview of the positioning of 16S rRNA clones in the small and large intestines and the available sequences of the Chinese *A. muciniphila* strains towards the *A. muciniphila-*type strain Muc^T^ and *A. glycaniphila* Pyt^T^. The percentages indicate the compositions of the clades. The tree was generated using the randomized axelerated maximum likelihood (RAxML) method version 7.0.3 in ARB using a 40% positional conservatory filter [42]. The original detailed maximum likelihood tree is shown in Supplementary Figure S1. Similar groups were observed using the neighbor joining method (Supplementary Figure S2). (**b**) Overview of Verrucomicrobia/*Akkermansia* sequences in the human digestive tract.

The human intestine may be colonized by different *Akkermansia*-like spp. [12]. The 16S rRNA sequences detected in metagenomic datasets suggested colonization by at least eight different *Akkermansia*-like spp. However, as these sequences derived from short Illumina reads, mis-assembly and other technical biases may have affected this conclusion. Moreover, it is also possible that simultaneous colonization by different species occurs. Recently, 39 *A. muciniphila* strains were isolated from human and mouse feces and subsequently analyzed for their 16S rRNA sequences and draft genomes [38,39]. All 16S rRNA sequences from these strains shared over 97% sequence identity with that of the type strain *A. muciniphila* Muc^T^ isolated from Europe, suggesting that they represent isolates from the same species. Using these isolates, three phylogroups (AmI, AmII, and AmIII) were identified based on core genome single nucleotide polymorphisms (SNPs). We constructed a maximum likelihood tree based on the available 16S sequences of the Chinese *A. muciniphila* strains in the National Center for Biotechnology Information (NCBI) sequence database and all other 16S rRNA *Akkermansia-*like sequences derived from colonic and ileal biopsy samples derived from the SILVA small subunit Reference 132 dataset (Figure 1a and Supplementary Figure S1). In this tree, the newly isolated Chinese *A. muciniphila* strains fall into two out of three distinct clades (clades one and two) that, however, have only minimal differences. In comparison with the type strain, the first clade has 99–100% identity, the second clade 98–99%, and the third clade 98%. Interestingly, the Chinese *A. muciniphila* strains have reportedly distinct metabolic and functional features [39]. However, unlike *A. muciniphila*-type strain Muc^T^, complete closed genomes of these Chinese *A. muciniphila* strains have not been reported. Furthermore, functional analysis of the Chinese *A. muciniphila* strains has not been performed, but the strains are able to grow on mucus [39]. Next to *A. muciniphila*, only one other *Akkermansia*-like sp. has been validly described, namely *Akkermansia glycaniphila* strain Pyt^T^ isolated from reticulated python feces (included in the phylogenetic tree shown in Figure 1a) [40,41]. The closest relative of *A. glycaniphila* is *A. muciniphila* Muc^T^, sharing 94.4% 16S rRNA sequence similarity [40]. The average nucleotide identity between the *A. glycaniphila* Pyt^T^ genome and the genome of *A. muciniphila* Muc^T^ was found to be 79.7%. Compared to *A. muciniphila*, *A. glycaniphila* is also able to use mucin as a sole carbon, energy, and nitrogen source. However, the relatedness between these two species, determined by DNA-DNA hybridization, is low, namely 28.3%. To be able to compare functional differences between Chinese *A. muciniphila* strains, the *Akkermansia*-like sequences found in biopsies of the ileum and large intestine (included in the tree), and *A. muciniphila* Muc^T^, enclosed genomes and functional analyses are needed.

Altogether, *A. muciniphila*’s presence varies among individuals. Its abundance varies not only from person to person but also from age group to age group. On top of that, other factors such as geographical location may also play an important role in the presence and richness of *Akkermansia*-like spp. in the human GIT.

## 3. Physiologic Adaptation of *Akkermansia muciniphila* to the Human GIT

*A. muciniphila* is an oval shaped, anaerobic Gram-negative bacterium that was first described by Derrien et al. [11]. Transmission electron microscopy revealed the presence of filamentous structures on Muc^T^ cells when grown on mucin medium. On top of this, strain Muc^T^ is able to exclude Indian ink, suggesting that the filamentous structures are capsular polymers. More recently, outer membrane proteins of *A. muciniphila* were analyzed, which resulted in the identification of pili proteins [13,25,26].

**Figure 2:**
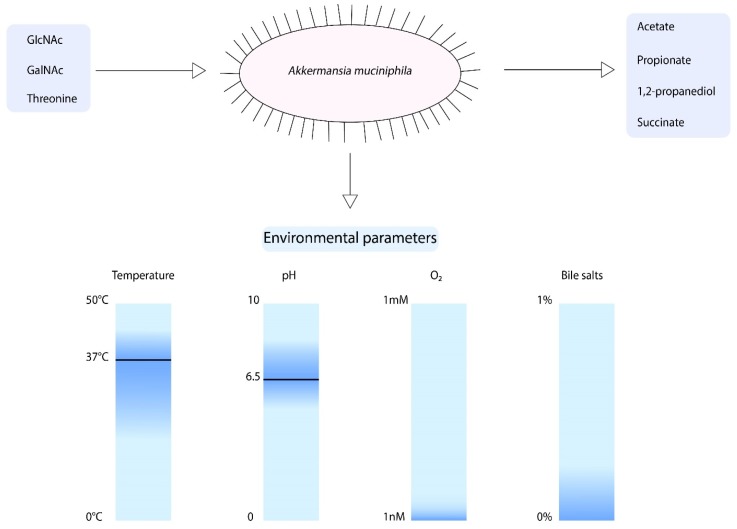
Schematic overview of the growth parameters of *A. muciniphila*. The optimum growth temperature and pH are 37 °C and 6.5, respectively. In addition, *A. muciniphila* is able to tolerate nM concentrations of oxygen and is able to grow in the presence of 0.1% purified bile salts.

When focusing on the growth parameters of *A. muciniphila*, it is known that growth was observed between temperatures of 20 and 40 °C and pH values of 5.5–8.0 (Figure 2) [11]. However, the optimum growth temperature and pH are 37 °C and 6.5, respectively. *A. muciniphila* is an obligate chemoorganotroph, utilizing mucus as a sole carbon, nitrogen, and energy source [11]. Consequently, the short chain fatty acids (SCFAs) acetate, propionate, and to smaller extent 1,2-propanediol and succinate are produced [11,43]. Another factor that influences the growth of *A. muciniphila* is the presence of oxygen. *A. muciniphila* was found to be able to tolerate and even benefit from nanomolar concentrations of oxygen in liquid medium [44]. Upon the presence of oxygen, there is a change of acetate to propionate production by *A. muciniphila*. This results in an increased production of ATP and NADH, which enhances the growth of *A. muciniphila*.

To compose a minimal medium for *A. muciniphila*, a genome-scale metabolic model was constructed [43]. This model predicts the degradation of mucin-derived monosaccharides. The model showed that *A. muciniphila* is able to synthesize all essential amino acids, except for l-threonine, was not present in the pathway. Furthermore, growth experiments revealed that *A. muciniphila* can degrade a variety of sugars such as glucose, *N*-Acetylglucosamine (GlcNAc), *N*-Acetylgalactosamine (GalNAc), and fucose. However, to obtain growth large amounts of casein tryptone, mucin or a rich medium was required. Hereafter, another study showed that *A. muciniphila* does not code for GlmS, which mediates the conversion of fructose-6-phosphate to glucosamine-6-phosphate [45]. This reaction is essential for peptidoglycan formation. Therefore, the degradation of glucose does not lead to biomass production. *A. muciniphila* does code for NagB, which catalyzes the reverse reaction, indicating that the addition of GlcNAc is essential for growth of *A. muciniphila*. Altogether, this information led to the development of a defined minimal medium for *A. muciniphila,* in which l-threonine and GlcNAc or GalNAc are essential components for growth [45].

Recently, the growth of *A. muciniphila* in the presence of bile has been studied [46]. Interestingly, the *A. muciniphila* abundance was positively correlated to circulating primary bile acids in mice [47]. The addition of 0.1%, 0.5%, and 1% porcine bile extract resulted in increased growth of *A. muciniphila* in comparison to the medium that did not contain bile [46]. In contrast, purified bile salts addition of 0.5% or higher resulted in inhibited growth of *A. muciniphila*, whereas the addition of 0.1% purified bile salts did not inhibit growth [48]. Moreover, the survival of *A. muciniphila* in gastric juice was found to be very low [46].

Several studies have described the antibiotic resistance of *A. muciniphila* [49,50]. The type strain of *A. muciniphila* (Muc^T^) was found to be susceptible to imipenem, piperacillin/tazobactam, and doxycycline, while resistance was noted for vancomycin, metronidazole, and penicillin G [49]. Another *A. muciniphila* strain was resistant against vancomycin and ofloxacin, but susceptible to penicillin, amoxicillin, ceftriaxone, and imipenem [50]. *A. muciniphila* MucT has potential beta-lactamase genes and may code for a 5-nitroimidazole antibiotic resistance protein [12]. The in silico prediction of a strain directly sequenced from stool (*A. muciniphila* strain Urmite) predicted the presence of antibiotic resistance genes for the classes beta-lactamases, glycopeptides, MLS (macrolides, lincosamides, streptogramines), phenicol, sulphonamide, tetracycline, and trimethoprim [51]. Guo et al. described the presence of three antibiotic resistance genes in *A. muciniphila* strain GP36, which originated from plasmid pRSF1010 (8684 bp) of *Salmonella enterica* [39]. This indicates that *A. muciniphila* might acquire antibiotic resistance genes through lateral gene transfer.

The growth parameters of *A. muciniphila* described above coincide with the environmental parameters found in the large intestine. The oxygen concentration in the intestine follows a steep gradient from the intestinal submucosa to the lumen, where the oxygen concentrations decrease to near anoxia [52]. *A. muciniphila* may take advantage of this oxygen concentration in the mucus layer, enhancing its growth. The mucosal layer of the large intestines serves as a carbon, nitrogen, and energy source for the use of *A. muciniphila*. The mucin backbone is rich in threonine (among other amino acids) and contains many sugar groups, including GlcNAc and GalNAc [53]. As mentioned earlier, these are among the minimal growth requirements of *A. muciniphila*. Although the mucus layer in the large intestine is thought to be the optimal niche for *A. muciniphila*, mucus is also present at other locations of the GIT. Several conditions may vary in the GIT, such as type of mucin that is secreted, pH, oxygen concentration, and concentration of bile acids. The ability of *A. muciniphila* to cope with these conditions will be discussed below.

## 4. *Akkermansia muciniphila* along the Human Gastrointestinal Tract

*Akkermansia*-like spp. were found to be present in different anatomical regions of the digestive tract, including the oral cavity, breast milk, pancreas, biliary system, small and large intestines, and appendix (Figure 1b). Next to the aforementioned regions, *Akkermansia*-like sequences were also detected in human blood [50,54,55]. However, the presence of *Akkermansia-*like sequences was only detected in subjects with diseases as septicemia and cirrhosis. In a mice study, *A. muciniphila* was detected in the oral cavity, stomach, small intestine, and large intestine the upon administration of breast milk and formula milk [56]. However, in this review we will mainly focus on the presence of *Akkermansia*-like spp. in the human body.

### 4.1. Oral Cavity

The oral cavity is a moist environment with a relatively constant temperature between 34 and 36 °C [57]. There are several ecological niches in the oral cavity that mostly have a neutral pH. The mean pH of the mucosal sites was found to be 6.78 ± 0.04 [58]. Examples of different sites in the oral cavity are the tongue, soft and hard palates, tooth surfaces, and tonsils [57]. In terms of temperature and pH, the oral cavity can support the growth of a wide variety of microorganisms. Therefore, the microbial community of the oral cavity is site-specific and highly diverse [59].

Several studies found a high abundance of the phyla Firmicutes, Proteobacteria, Bacteroidetes, and Actinobacteria, with *Streptococcus* (belonging to the phylum Firmicutes) being the most abundant genus [60,61]. The presence of Verrucomicrobia in the oral cavity is often not described [60,61,62,63,64]. However, the presence of *Akkermansia*-like sequences was found in the oral cavity of a choledocholithiasis patient [65]. The salivary samples were taken by gargling with 20 mL of sterile saline water. Thus, the microorganisms in these samples originate from multiple niches in the oral cavity.

The relative abundance of *Akkermansia*-like spp. in this sample was low, namely 0.02% [65]. In addition, these sequences were only found in one out of six patients included in this study. In terms of pH and temperature, *Akkermansia* spp. could be able to survive in the oral cavity [11]. Furthermore, Gram-negative and obligate anaerobes with proteolytic lifestyles are present in healthy gingival crevice biofilms [66]. As discussed before, *A. muciniphila* has a mucin-degrading lifestyle [11]. The mucins found in the oral cavity are MUC5B, MUC7, MUC19, MUC1, and MUC4 [67]. Of these mucin structures, MUC5B is the most abundant gel-forming mucin in the oral cavity [68,69]. As *A. muciniphila* has mucin-degrading enzymes similar to those found in oral *Streptococcus* spp., *A. muciniphila* might be able to use the mucin structures in the oral cavity as a substrate for growth [70,71]. However, further research is needed to confirm this hypothesis.

Mucin oligosaccharides are able to bind microbes and in some cases exert functions in antimicrobial activity or carry antimicrobial proteins [67]. For example, MUC7 and MUC5B both bind statherin and histatin-1. By binding these molecules, mucins exert a protective function in the oral cavity. The potential role of *Akkermansia* spp. in the oral cavity is unknown. However, one could hypothesize that its capability to stimulate the mucus production of the epithelial cells enhances their protection e.g., against pathogens. Another possibility could be that *Akkermansia* spp. produces compounds in mucin degradation that could be useful for other bacteria in the oral microbial community. Lastly, *Akkermansia* spp. might be involved in the modulation of the host response. Altogether, more studies should be conducted to confirm the presence of *Akkermansia* spp. and its function in the oral cavity.

### 4.2. Pancreas

The pancreas is a complex organ comprised of both exocrine glands (secreting digestive enzymes into the intestinal lumen) and endocrine glands, called the islets of Langerhans, which secrete hormones directly into the bloodstream [72]. The pancreas plays a central role in human metabolism, allowing ingested food to be converted and used as fuel by cells throughout the body. The pancreas may be affected by devastating diseases, such as pancreatitis, pancreatic adenocarcinoma (PAC), and diabetes mellitus (DM), which generally results in a wide metabolic imbalance [73]. Nutrient metabolism in pancreatic cells is not only essential for providing energy for the cell, but also serves as a mechanism to sense and react to circulating levels of macronutrients. This gives the pancreas a central role in metabolism regulating the whole-body energy homeostasis. Efficient energy metabolism in pancreatic endocrine cells of the islets is required to permit the secretion of many hormones, mainly insulin and glucagon, that regulate glucose and lipid utilization throughout the body. The pancreas is thought to be devoid of bacteria. However, microbial translocation as a result of disease states has led to measurements of microbes in pancreatic tissue. Because it is dangerous and impossible to take biopsies of pancreatic tissue, due to the risk of leakage of pancreatic fluid, pancreatic samples can only be obtained via surgery. As such, samples of healthy individuals are not yet reported. Healthy controls are usually healthy tissue surrounding a pathologic site. Recent research has shown that disruption of pancreatic metabolism is often a consequence of disruptions in the gut microbiome [74]. Another study has shown that the pH in the pancreas drops significantly (*p*-value < 0.05) in patients with painful chronic pancreatitis (7.02 ± 0.06) compared to healthy individuals (7.25 ± 0.04) [75]. That pH change in patients with chronic pancreatitis might be one of the reasons why the bacterial barrier in the pancreas is ruptured and the abundance of bacterial phyla and species is elevated [76].

*A. muciniphila* plays an important role in the maintenance of the gut barrier function [16]. A few studies have shown the association of *A. muciniphila* with the pancreas and its health. A recent study with patients undergoing pancreatic fluid pancreaticoduodenectomy (PD) revealed that the mean relative abundances of the Verrucomicrobia phylum and *Akkermansia* genus, respectively, were 0.0005 and 0.0004 in the pancreas of these patients [77]. The study also highlighted that in the pancreas tissue of the patients, other commensal bacteria were found to be present. The Proteobacteria phylum and *Klebsiella* genus were the most abundant, with mean relative abundances of 0.5410 and 0.2011, respectively. *Faecalibacterium*, *Bacteroides*, and *Prevotella* were also detected in the pancreas of PD patients. The presence of other gut microbes in the pancreas apart from *Akkermansia* may indicate trophic interactions between them.

Interestingly, it was found that mice treated with pancreatic enzyme replacement therapy (PERT), had a significant, 58-fold increase of *A. muciniphila* sequences compared to the control samples (mice treated with tap water) [78]. Furthermore, it is stated that pancrealipase diminishes pancreatic exocrine insufficiency-associated symptoms by inducing the colonization of *A. muciniphila* followed by the normalization of the intestinal barrier. Therefore, it is hard to speculate on the role of *Akkermansia* species and/or *A. muciniphila* in the pancreas.

The *Akkermansia* genus is detected in the pancreas, mainly in conditions of pathology. The significant change of pH of the pancreatic fluid in patients with chronic pancreatitis (7.02 ± 0.06) might be a reason why *Akkermansia* was detected in pathological conditions. Thus far, there is not any evidence showing that the pancreas is colonized by bacteria in healthy individuals or that it is a bacteria-free organ. In all of the studies that have been described so far, the bacterial colonization of the pancreas is inextricably linked to the cause of pancreatic disease. The higher abundance of bacteria in patients with pancreatic diseases could be associated with an overall higher abundance of microbiota members in these states of disease due to bacterial overgrowth and translocation. Decreased gut barrier function in both acute and chronic pancreatitis increases bacterial translocation. This bacterial translocation could have significant impact on the nutrient absorption and therefore on the availability of nutrients for intestinal microorganisms, and the microbial composition of the gut.

### 4.3. Bile Ducts and Gallblader

Bile is a complex aqueous solution secreted by the liver [79]. Both gallbladder bile and common bile duct bile of patients undergoing cholecystectomy were found to have an alkaline pH in the ranges of 6.8–7.65 and 7.5–8.05, respectively [80]. In most animal/mammal species, bile contains less than 5% solid contents [79]. The most abundant organic substances in bile are bile salts [81]. The primary bile salts in the mammalian liver are cholic acid and chenodeoxycholic acid (CDCA). They are produced from cholesterol in the liver and are then excreted into the duodenum [82]. Bile salts exert several functions. For example, they were found to have a role in antimicrobial defense, promoting lipid absorption and protein digestion and assimilation [83,84]. Conjugation of these bile salts occurs at the side chain, where either taurine or glycine is added, leading to the formation of stronger acids [81]. Intestinal bacteria are able to convert the stronger acids producing secondary bile acids by deconjugating them. Large amounts of bile salts are secreted into the intestine; however, only limited amounts are excreted from the human body [85]. More than 95% of the bile salts are reabsorbed in the ileum and redirected to the liver for recirculation. According to this enterohepatic circulation mechanism, each bile salt is recirculated approximately 20 times.

Interestingly, the bile duct was first considered to be generally sterile [86]. However, more recently several studies have focused on the microbial community present in bile [65,77,87,88,89,90]. The phyla Firmicutes, Fusobacteria, Proteobacteria, Actinobacteria, and Bacteroidetes (among others), have been identified in bile samples [65,77,88,89]. These phyla are all commonly found in the human GI tract [91]. Subsequently, the bacteria found in the biliary tract are likely to originate from the human duodenum [65]. The most common genera in bile samples were found to be *Prevotella*, *Streptococcus*, *Veillonella*, *Fusobacterium*, and *Haemophilus* [89].

To study the function of the biliary microbiota, predictive functional profiles were constructed using Phylogenetic Investigation of Communities by Reconstruction of Unobserved States (PICRUSt) [65]. This method revealed that biliary bacteria have significantly enriched pathways, in comparison to the upper digestive tract microbiota, related to environmental information processing, cell motility, carbohydrate metabolism, amino acid metabolism, and lipid metabolism. Furthermore, several studies have focused on the role of the biliary microbiota in the development of diseases such as gallstone disease and biliary neoplasia in primary sclerosing cholangitis (PSC) [88,89,92,93]. However, to explore the exact role of the microbiota in gallstone formation, more research needs to be conducted. In addition, PSC was not associated with changes in the microbial community of the biliary system [89]. However, *Streptococcus* species were found to be positively correlated to disease progression and might therefore have a pathogenic role in the progression of PSC.

Although the presence of Verrucomicrobia and/or *Akkermansia*-like sequences has not been described in all studies involving the biliary microbiota, they have been found to be present in a proportion of studies including bile samples [65,77,88,89]. In a study describing the bacterial community in bile and gallstone samples, *Akkermansia*-like sequences were detected [88]. The relative abundance of *Akkermansia*-like sequences in 12 out of 26 bile samples ranged between 0.03 and 0.4%. In addition, 10 out of 29 gallstone samples contained *Akkermansia* spp. with a relative abundance ranging between 0.02% and 0.3%. *Akkermansia*-like sequences were also detected by another study in the bile sample of one out of six gallstone patients, revealed using V3-4 Illumina sequencing [65]. The relative abundance of *Akkermansia*-like sequences in this sample was low, namely 0.153%. In addition, the mean relative abundances of Verrucomicrobia and *Akkermansia* spp. were determined to be 0.05% and 0.04%, respectively, in bile samples from 50 patients undergoing pancreaticoduodenectomy [77]. Furthermore, bile samples of subjects with opisthorchiasis (bile duct infection by *Opisthorchis felineus* [94]) contained higher amounts of Verrucomicrobia (among other phyla) than subjects with gallstone disease without infection of *O. felineus* [93]. The presence of Verrucomicrobia was not specific to the *Akkermansia* genus and the exact relative abundance of Verrucomicrobia was not provided. Even though five phyla including Verrucomicrobia were found to be more abundant in infected subjects than non-infected subjects, there were no functional differences between these groups based on analysis using PICRUSt. The studies described in this section were properly controlled for contaminants.

The pH values of gallbladder bile and common bile duct bile are both within the growth range of *A. muciniphila*. However, for *Akkermansia* spp. to be able to remain in bile, they are expected to harbor a mechanism for protection against bile. A putative bile acid transporter gene (Amuc_0139) is annotated in the genome of *A. muciniphila*, which might function to export bile acids from the cell [48]. This process could reduce the effects of bile acids inside the cells of *A. muciniphila*. Another possible mechanism, as identified in *Bifidobacterium* spp*.*, is the protection of the cell wall against bile acids by the production of exopolysaccharides [95,96]. However, this mechanism has not been identified for *Akkermansia muciniphila.*

Several mucins have been identified in the biliary tract, specifically in the gallbladder. The mucins that are expressed in the gallbladder are MUC3, MUC5B, MUC5AC, and MUC6 [97,98,99,100]. Even though colonic mucin consists mainly of MUC2, *A. muciniphila* might be able to use the mucins in the gallbladder as a substrate. Next to *Akkermansia* spp. other bacteria with mucin-degrading capacities in the GI tract have been identified in the biliary system. As such, *Streptococcus anginosus* [93] and bacteria with operational taxonomic unit (OTU) IDs with 99% identity matching that of *Bacteroides vulgatus* [88] were found to be present in bile samples. Although the function of *Akkermansia* spp. in the biliary system is unknown, it might have a role in the strengthening of the mucosal barrier. In this way, the mucosal layer may provide increased protection against pathogens. This could explain the increase of Verrucomicrobia during infection of *Opisthorchis felineus,* since it might function to strengthen the mucosal barrier and thereby provide protection during infection.

### 4.4. Small Intestine

The GIT supplies the human body with energy and essential nutrients [101]. This is achieved by the conversion and absorption of food components reaching the small intestine. The small intestine can be divided into three segments: duodenum, jejunum, and ileum [102]. The transit time of the small intestine was found to be between 30 min and 4.5 h [103]. Once the gastric content enters the duodenum, it is neutralized by bicarbonate derived from the pancreas, liver, and duodenal mucosa, causing pH fluctuations [104]. More recent investigations of pH profiles revealed that pH values increased from 5.9–6.3 in the proximal part of the small intestine (duodenum) [105]. In the distal parts, pH values were found to increase to pH 7.4–7.8.

The epithelial cells within the small intestine are covered with mucus. In contrast to the mucosal layer in the stomach and the colon, the mucosal layer in the small intestine is thinner and less dense [106]. In addition, this layer is not firmly attached to the epithelial surface and forms a soluble mucus gel [106,107]. The mucus gel layers observed in the duodenum and jejunum are similar in thickness, although no loose/sloppy mucus was found in the jejunum [106]. In contrast to the duodenum and jejunum, the mucus layer in the ileum is significantly thicker. The accumulation rates were similar throughout the different segments of the small intestine.

The fast transit time, in comparison to the large intestine, contributes to limited microbial growth in the small intestine [102]. In addition, the secretion of digestive enzymes and bile into the small intestine creates a harsh environment in terms of microbial growth [101]. The bacterial concentration in the duodenum and jejunum is only 10^3^–10^4^ bacteria/mL content [108]. This concentration increases in the ileum, where the bacterial concentration is 10^8^ bacteria/mL content. Due to the challenging conditions for microbial growth in the small intestine (acidity and higher oxygen levels than in the colon), the microbial community is dominated by bacteria that are facultative anaerobic, able to grow quickly, and able to tolerate bile acids and antimicrobials [109]. At the same time, these bacteria are also competing with the host and other microorganisms for simple sugars in the small intestine. Interestingly, phagocytes in the small intestine are thought to play a role in immune surveillance of the small intestinal mucosa [110]. This means that phagocytes are able to selectively take up bacteria, which might be linked to maintaining immune homeostasis.

The location of the small intestine in the human body causes difficulties in sampling, in comparison to, for example, the oral and fecal microbiota. Therefore, fewer studies have been performed describing the microbiota in the small intestine [101,111]. In the duodenum, the phyla Firmicutes and Actinobacteria were found to be predominant in the duodenal fluid of both obese and healthy groups (*n* = 5 for each group) [112]. Other (less abundant) phyla detected in the duodenum were Proteobacteria, Fusobacteria, TM7, Bacteroidetes, and Tenericutes. However, an inter-individual variability in the taxonomic composition between these samples was observed. Even though the duodenum was found to have fewer OTUs than mouth, colon, and stool samples, it does harbor most phyla observed in the other sites [91]. The mucosa-associated microbiota of the duodenum was found to be dominated by the phylum Firmicutes and genus *Streptococcus* [113]. The genera *Prevotella, Veillonella*, and *Neisseria* were also found to be present in the mucosal layer. Interestingly, the duodenal mucosa-associated microbiota found in this study overlaps in broader levels of classification with that of the oral cavity and saliva. Further down the small intestine, the most dominant phyla in jejunal fluid were found to be Firmicutes, Proteobacteria, and Bacteroidetes [111]. Less abundant phyla (5–10%) were Actinobacteria and Fusobacteria. In comparison to the findings of the microbiota composition in the duodenum, the abundance of Proteobacteria and Bacteroidetes were found to be dominant over Actinobacteria in the jejunum [111]. When comparing the microbiota composition found in samples obtained after the washing procedure and mucosal biopsies, these compositions were highly similar. The last part of the small intestine (ileum) was found to be dominated by the phyla Bacteroidetes, *Clostridium* cluster XIVa, and Proteobacteria [101,114].

Several studies have focused on the small intestinal microbiota in disease states, such as IBS, Crohn’s disease, and liver cirrhosis [115,116,117,118,119]. A study focused on IBS showed that the small intestinal microbiota of IBS patients and healthy individuals did not differ in terms of major phyla or genera [117]. In contrast, the duodenal mucosal microbiota of cirrhotic patients were surprisingly different to that of healthy controls [118]. The dysbiosis observed in duodenal samples of cirrhotic patients might be associated with an altered oral microbiota or an altered environment of the duodenum.

Even though the phylum Verrucomicrobia is not included in the dominant microbial compositions described above, Verrucomicrobia and *Akkermansia*-like spp. have been detected in all segments of the small intestinal tract. In duodenal fluid, Verrucomicrobia and *Akkermansia*-like spp. were found in three out of six subjects (with relative abundances of 0.17%, 0.012%, and 0.013%) using V3-4 Illumina sequencing [65]. In addition, Verrucomicrobia (not specified to *Akkermansia*) were detected in duodenal biopsies (0.0688%) and mucus (0.0387%) using 454/Roche GS FLX sequencing [120]. Jejunal contents have also been shown to harbor *Akkermansia*-like spp., with a mean relative abundance of 0.01% (*n* = 17) [77]. Analysis of swabs from jejunal contents were performed using Illumina MiSeq. Another study detected *Akkermansia*-like spp. in four out of 20 subjects with concentrations ranging from three to 90 hits, equaling to 0 to 0.029% of total hits, also using Illumina MiSeq [77]. Furthermore, Verrucomicrobia were found in the distal ileum using direct cloning and sequencing, making up 5% of the detected microbial community [121]. Lastly, *Akkermansia*-like sequences were detected in the ileocecal biopsies of patients with PSC and ulcerative colitis, as well as non-inflammatory controls (relative abundances of 0.49% ± 0.52%, 0.37% ± 0.37%, and 0.36% ± 0.31%, respectively) [122]. In the schematic tree in Figure 1a, the *A. muciniphila* sequences derived from ileum biopsies are clustered together. This occurs in particular in the third clade, which is made up entirely of *Akkermansia*-like sequences derived from the ileum, with a 16S rRNA sequence identity of 98% in comparison to *A. muciniphila* Muc^T^. The isolation of *Akkermansia*-like species in the ileum is needed to study possible differences between the strains found in the small intestine and the strains in the large intestine. Considering the pH in the small intestine, the *Akkermansia*-like spp. found in the ileum and other parts of the small intestine could have a different optimum pH for growth.

The function of the small intestinal microbiota was also studied using comparative metagenomics and RNAseq [101]. This study, in which *A. muciniphila* was not detected, revealed that the metabolic focus small intestinal microbiota lies within carbohydrate uptake and metabolism. In more detail, simple carbohydrate transport phosphotransferase systems, fermentation, central metabolism, the metabolism of amino acids, and the production of cofactors were enriched. A more recent study emphasized that the ileum mucosal microbiota might have a role in plant cell wall polysaccharide (PCW) degradation [123]. A large portion of the glycans that reach the small intestine are PCW polysaccharides. These polysaccharides cannot be degraded by humans, whereas the ileal microbiota associated with the mucus layer was found to have the enzymatic potential to break down PCW polysaccharides. The exact role of *A. muciniphila* in the small intestine remains unknown, but *Akkermansia*-like spp. could have a role in immune signaling in this part of the GIT. In mice, the addition of *A. muciniphila* in comparison with germ-free mice resulted in more significant modulation in the ileum of PPARα-RXRα activation, tryptophan metabolism, serotonin receptor signaling, and dopamine receptor signaling, among others [20]. The number of differentially expressed genes in the ileum of *A. muciniphila* mono-associated mice was 253 (144 upregulated and 99 downregulated genes). The administration of *A. muciniphila* resulted in an increase of Reg3g expression under the control diet and a decrease of Lyz1 expression in the ileum [10]. Another study also showed a decrease in Cnr1 expression and increase of Cldn3 expression in the ileum upon the administration of *A. muciniphila* in mice [124]. As discussed before, the mucus layer in the small intestine is thinner and less dense than that of the colon [106]. This allows closer contact between the microbial community and host cells, promoting immune signaling in this region of the gastrointestinal tract. The presence of *Akkermansia*-like spp. in the ileum might contribute to immune health.

### 4.5. Large Intestine

The large intestine specializes in digestion and consists of several different segments, namely the cecum, ascending colon, transverse colon, descending colon, rectum, and anus [125]. The colonic transit time of healthy individuals is longer than the transit time of the small intestine, ranging between 9 and 46 h (mean 28 h) [126]. In healthy individuals, a decrease in luminal pH is observed in the cecum in almost all subjects (pH ranging from 5.5–7.5) [127]. This drop in pH is due to the fermentation of carbohydrates by colonic bacteria, leading to the production of SCFAs. Then, the pH increases along the large intestines to pH values ranging between 6.1 and 7.5. The mucosal pH of the large intestine parallels the luminal pH [128]. However, the mucosal pH is less acidic than the luminal pH in all anatomic regions of the large intestine.

The epithelial cells along the large intestine are covered by the mucosal layer [129]. As such, the mucosal layer protects the epithelial cells from direct contact with microorganisms. On top of this, the mucosal layer also contains antimicrobial proteins such as IgA. The mucosal layer can be divided into two parts: the inner and outer mucus layers. The inner mucus layer is firmly attached to the epithelial cells and devoid of bacteria, whereas microbes are capable of colonizing the outer layer due to its higher permeability [107]. Both layers are mainly composed of the gel-forming mucin MUC2, consisting of large polymers that are formed by N-terminal trimerization and C-terminal dimerization [130]. The expansion of the mucus layer occurs due to increased pH and decreased calcium (Ca^2+^) levels. N-terminal interactions are weakened by the decreased calcium concentrations. Therefore, water is able to bind to the mucin domain glycans, leading to the formation of flat mucin sheets. Furthermore, the less dense outer layer is the result of endogenous proteases, promoting the possibility of microbes to colonize the mucus layer [107]. The degradation of these mucins by mucin-degrading bacteria of the colon microbiota affects the host cells e.g., by producing SCFAs [131].

The gut microbiota is mainly studied using fecal samples, since these samples can be obtained without colonoscopies. However, microbial communities detected in fecal samples mainly reflect the luminal microbiota in the distal large intestine. Therefore, the microbial communities in biopsies can differ distinctly from that in fecal samples [91]. Several studies have confirmed that the microbial communities in biopsy samples of different anatomic regions of the colon show similarities, focusing on the major phylogenetic groups [91,121,132]. Along the intestinal tract, Firmicutes and Bacteroidetes were predominant, with lower proportions of Proteobacteria and Fusobacteria observed in biopsy samples [91,132]. The sequencing of fecal samples of 22 individuals from four different European countries revealed the presence of three robust clusters, called enterotypes [133]. These enterotypes are either enriched in (1) *Bacteroides*; (2) *Prevotella* and co-occurring *Desulvibrio*; or (3) *Ruminococcus* and co-occurring *Akkermansia*. Recently, a method of restricting enterotyping space was proposed to increase the ability to detect samples outside of these enterotyping spaces [134]. Overall, the dominant phyla in fecal samples derived from healthy individuals were found to be Firmicutes, Bacteroidetes, and Actinobacteria. Less abundant phyla were Proteobacteria and Verrucomicrobia [133].

In addition to studies on the role of the gut microbiota in health, the gut microbiota has also been studied in diseases such as IBD, IBS, obesity, and type-2 diabetes. A shift in microbiota composition was observed in IBD patients and may have a role in the onset, maintenance, and severity of the disease, although this shift could also partly be due to the disturbed gut environment [135]. In IBS patients, a decrease in bacterial diversity was observed [136,137]. However, a consistent gut microbiota pattern in IBS patients is lacking [138]. In obesity, inconsistent findings have been reported. For example, one study reported an increase of Firmicutes and a decrease of Bacteroidetes, while another reported the opposite [139,140]. Lastly, a decrease in canonical butyrate-producing bacteria was found in patients with T2D [141]. A decrease in butyrate-producing bacteria was associated with an increase in opportunistic pathogens, mainly Proteobacteria. Taken together, the studies on the gut microbiota in individual diseases are not all uniform, highlighting the difficulties in appointing markers for disease.

The gut microbiota plays an important role in the metabolism of host nutrients and the health maintenance of the host. Carbohydrates (mainly polysaccharides) that have not been hydrolyzed in the small intestine become available for the microbial community in the colon [142]. The main substrates entering the colon are resistant starch and polysaccharides derived from plant cell walls. The major end products produced by the gut microbiota are SCFAs (e.g., acetate, propionate, and butyrate) and gases (e.g., H_2_ and CO_2_) using the available substrates. Of these, butyrate is used as an energy source by colonic epithelial cells [143]. Furthermore, propionate is able to signal to the host through the GPR41 and GPR43 receptors [144]. Interestingly, SCFAs activate free fatty acid (FFA) receptor 2 and FFA3 in the gut [144,145,146]. These receptors control peptides (peptide YY and glucagon-like peptide 1) involved in appetite regulation [147]. Therefore, SCFA production in the gut may be associated with food intake. Next to dietary carbohydrates, the colonic microbiota also has a role in the degradation of host-derived glycans (mucin), xenobiotics, and drugs [142,148]. The gut microbiota is also able to stimulate host immunity in order to protect the host against pathogens [149]. In this way, the gut microbiota enhances the innate immune response and has a role in increasing the gut barrier function. One of the microbial species in the gut involved in immune regulation and increasing gut barrier function is *A. muciniphila* [10,26].

A recent study revealed the presence of Verrucomicrobiae in all anatomical regions of the large intestine by sequencing the V2 region [34]. The concentrations of Verrucomicrobiae (in two individuals) ranged between 0.3% and 15.8%. Interestingly, one individual harbored only low concentrations, ranging between 0.3% and 1.4%, while concentrations in another individual ranged between 9.8% and 15.8% throughout the anatomic regions. Notably, the individual with higher Verrucomicrobiae concentrations was a Crohn’s disease patient. Differences were not only observed between individuals, but also between the experimental designs. The use of another DNA extraction method resulted in lower amounts of Verrucomicrobiae with concentrations in the range of 0.3–7.3% in both individuals. Next to this study, there are more studies identifying the Verrucomicrobia phylum and/or *Akkermansia-*like spp. focusing on several anatomic regions of the large intestine [91,121,150,151,152,153]. Verrucomicrobia were identified in the cecum by another study, although quantities were not shown [150]. Even though the pH is lower in the cecum (pH 5.5–7.5), it is still within the growth range of *A. muciniphila*. In the ascending colon, Verrucomicrobia were identified with a concentration of 6% in the large intestine of a healthy volunteer [121]. Using a qPCR approach within the same region, a concentration of 4.17 ± 0.6 log 10 genomes per gram of a sample of *Akkermansia*-like spp. was described [151]. Furthermore, the transverse colon of two out of four included subjects showed the presence of Verrucomicrobia, with 563 and 7771 sequence counts of this phylum in each sample [91]. In the sigmoid colon, compared to the transverse colon, the same study reported a decrease in one of the subjects (from 563 to 64 sequence counts), whereas an increase was noted in another subject (from 7771 to 11,941 sequence counts). The qPCR approach resulted in a similar concentration to that found in the ascending colon, namely 4.16 ± 0.56 log 10 genomes per gram of a sample [151]. Several studies have also described the presence of *Akkermansia-*like sequences in the rectum [91,121,152]. Where one study reported a higher concentration in the rectum (9%) than in the ascending colon, another reported a rapid decrease in sequence counts from 64 and 11,941 in the sigmoid colon, to sequence counts of 1 and 2, respectively [91,121]. In conclusion, based on these studies, the presence and abundance of *A. muciniphila* in the large intestine is subject-specific.

To compare the 16S rRNA *Akkermansia*-like sequences in biopsies to fecal samples, a maximum likelihood tree was constructed (Figure S3). Interestingly, the majority of the *Akkermansia*-like sequences derived from biopsies were clustered together. This cluster contains a low amount of *Akkermansia*-like sequences derived from fecal samples. Therefore, one could hypothesize that sub-populations of *Akkermansia* spp. exist within the large intestine. However, it should be noted that complete *Akkermansia* spp. genomes derived from biopsies and lumen are needed to support this hypothesis.

In contrast to the presence of *A. muciniphila* in other regions of the GIT, its function has been more explored in its ecological niche. *A. muciniphila* was found to be correlated to health and inversely correlated to several disease states (as explained in the introduction). However, besides its presence in health and disease, *A. muciniphila* was also found to be involved in syntrophic interactions [154]. For example, co-cultivations of *A. muciniphila* with butyrate-producing bacteria (*Anaerostipes caccae, Eubacterium hallii*, and *Faecalibacterium prausnitzii*) resulted in butyrate production. Therefore, it is suggested that the mucus-degrading capacity of *A. muciniphila* stimulates intestinal metabolite pool and specifically butyrate levels, which is beneficial for the host. Another example is the release of sulfate during mucin degradation. This sulfate might be used by sulfate-reducing bacteria in the colon, producing hydrogen sulfide [155,156]. In turn, *A. muciniphila* predictively harbors genes involved in l-cysteine biosynthesis using hydrogen sulfide, suggesting that *A. muciniphila* might have a role in the detoxification of hydrogen sulfide in the intestines [157].

As mentioned earlier, the colonization of *A. muciniphila* in mice led to an increased expression of genes associated with immune responses and the strengthening of the gut barrier function [10,20]. In addition, the outer membrane protein (Amuc_1100) was found to be involved in immune regulation and the enhancement of trans-epithelial resistance [26]. Altogether, these studies suggest an important role of *A. muciniphila* in the microbial community of the large intestine in addition to its role in host interactions, promoting the use of this bacterium as a therapeutic agent for intestinal disorders.

### 4.6. Appendix

The human appendix extends from the cecum and is 5–10 cm long and 0.5–1 cm wide [158]. The function of the appendix has been up for debate for quite some time. Charles Darwin described the lack of function of the appendix and noted that the appendix is a remainder from primate ancestors that ingested leaves, in which the appendix functioned to ferment plant material [159,160]. The appendix is covered in gut-associated lymphoid tissue, suggesting its involvement in immune function [161]. An apparent function of the human appendix was described, suggesting that the appendix functions as a “safe house” for beneficial bacteria [158]. This same study revealed a higher abundance of microbial biofilms in the appendix than other areas of the human colon. To our knowledge, a description of the pH in the human appendix is lacking. However, the rabbit appendix was found to have a pH ranging between 6.2 and 6.7 [162]. Secretions of the rabbit appendix are rich in bicarbonate and occur spontaneously and at a relatively rapid rate (1–12 mL/h). Therefore, it has been suggested that in rabbits, the appendix may have a major role in the regulation of pH in the cecum. However, similar data is not available for the human appendix.

Considering the difficulty of obtaining samples of the human appendix, there are few studies describing the microbiota of the appendix. These studies mainly focus on the microbiota in appendicitis in comparison to healthy controls [163,164,165]. In healthy controls, the taxa *Fusibacter*, *Selenomonas,* and *Peptostreptococcus* were increased in comparison to the rectal microbiota [164]. This finding indicates that the human appendix harbors a distinct microbiota. A wide variation of abundances in phylum, genus, and species levels was detected within groups divided by health and severity of inflammation [165]. In healthy controls, Firmicutes and Bacteroidetes were found to be the most abundant phyla. Other abundant phyla detected in these samples were Fusobacteria, Actinobacteria, and Proteobacteria. Phyla with lesser abundances (<2%) were Spirochaetes, Cyanobacteria, Synergistetes, Tenericutes, and Verrucomicrobia.

While some studies found significant differences between the microbiota of appendicitis patients and healthy controls [163,164] or between severity of inflammation [166], another did not [165]. One of the genera linked to appendicitis is *Fusobacterium* [163,166]. Increased abundances of this genus were observed in appendicitis patients in comparison to healthy controls [163], and the presence of *Fusobacterium* could be linked to the severity of inflammation [166]. In contrast, the presence of *A. muciniphila* was found to be inversely correlated to the severity of appendicitis [14]. Using fluorescence in situ hybridization (FISH), the mean proportion of single bacterial groups for *A. muciniphila* was found to be 4.0 ± 4.6, 1.0 ± 2.1, and 0.2 ± −0.6 for no appendicitis, catarrhal appendicitis, and suppurative appendicitis, respectively.

The mucus layer of the appendix was found to contain a more concentrated biofilm than other parts of the large bowel [158]. Therefore, the appendix might be a favorable niche for mucin-degrading bacteria, including *A. muciniphila*. Although the role of *A. muciniphila* in the appendix is not specified, one could hypothesize that, being part of the appendiceal microbiota, *A. muciniphila* might have a role in re-colonizing the colon after an infection or colonic dysfunction. Thereby, *A. muciniphila* could function in the maintenance of a healthy gut microbiota by restoring the mucus barrier function subsequent to infection/inflammation.

### 4.7. Human Breast Milk and Early Life Intestine

In breast-fed infants, the main source of glycans are human milk oligosaccharides (HMOs) [167]. Human milk consists of a mixture of nutrients for infants, conveying immunologic and other health benefits [147]. Human milk contains 5–15 g/L HMOs, and over 200 different HMO structures exist. The major monosaccharides present in HMOs are d-glucose, d-galactose, *N*-acetyl-glucosamine, l-fucose, and *N*-acetylneuraminic acid (sialic acid) [168]. HMOs in the infant gut act as substrates for specific bacteria in the gastrointestinal tract, functioning as natural prebiotics by stimulating the growth of beneficial intestinal bacteria such as bifidobacteria and lactobacilli [168,169]. It is remarkable to mention that milk oligosaccharides and glycoconjugates are able to prevent the development of pathogens and toxins, inhibiting their binding on the surface of the epithelial cells [170]. The structure of HMOs has chemical similarities to mucus glycans [171].

*A. muciniphila* has been identified in human milk samples immediately after delivery (colostrum), and at 1 and 6 months [172]. *A. muciniphila* cell counts in breast milk were measured after conducting qPCR, revealing that *A. muciniphila* was higher in abundance in overweight than normal weight mothers, with mean concentrations of 1.25, 1.09, and 1.20 log number of gene copies/mL in colostrum samples and breast milk samples. Furthermore, *A. muciniphila* was observed to be present in colostrum samples that were collected from 11 women after elective caesarean with a median counts number of 0.9 (interquartile range from 0.0 to 1.5) analyzed by qPCR [173]. In turn, in samples from human breast tissue from 43 women (aged 18 to 90 years), the presence of *Akkermansia*-like species was found using 16S rRNA sequencing [174].

As mentioned earlier, *A. muciniphila* is also present in infants’ intestines from the first months of life [27,29,30]. The structures in HMOs can also be found in mucus glycans [175,176]. *A. muciniphila* was able to break down structures of HMOs into simpler sugars, releasing SCFAs (acetate and propionate) in the media. *A. muciniphila* expressed enzymes that were involved in carbohydrate and glycan degradation, such as α-l-fucosidases, exo-α-sialidases, β-galactosidases, and β-hexosaminidases [177]. This indicates that *A. muciniphila* might be able to use HMOs, using human milk as a sole energy, carbon, and nitrogen source, which could also explain its presence in breast milk and the breast tissue of lactating woman.

To confirm this, further research should be conducted to gain more insight in the mucolytic activities and the function of *A. muciniphila* in human milk. The presence of *Akkermansia* spp. and *A. muciniphila* specific in human milk may benefit the maturation of the infant’s microbiota establishment and immune maturation, as its outer protein was found to be involved in immune regulation [26]. Last but not least, *A. muciniphila* glycan degradation ability might be proved to play an important role in the initial colonization of the infant’s gut, thus having a major impact on later life.

### 4.8. Akkermansia Muciniphila in In Vitro Gut Models

In contrast to invasive sampling of the human body, in vitro models have also been introduced to study the spatial organization of the human gut microbiota. Multiple in vitro models are available for this purpose, such as the Gastro-Intestinal Model (TIM-1 and TIM-2) and the Simulator of Human Intestinal Microbial Ecosystem (SHIME). The small intestinal model TIM-1 consists of four compartments representing the stomach, duodenum, jejunum, and ileum [178], while TIM-2 simulates the large intestine [179]. The SHIME was developed in 1993 [180]. This model simulates five compartments of the digestive tract, namely the stomach, small intestine, and the ascending, transverse, and descending colon. In addition, a variation on SHIME was developed, named the mucosal SHIME (M-SHIME) [181]. The M-SHIME has a mucosal compartment, developed to study the microbial colonization of the mucus layer.

Most of the studies including *A. muciniphila* used the SHIME model. The presence of *A. muciniphila* in different compartments of in vitro models has also been evaluated [182,183]. In SHIME, *Akkermansia* spp*.* are more abundantly present in the transverse and descending colon compartments than in the proximal compartments (ascending colon) of this model [182,183,184,185]. This is not in concordance with findings using biopsy samples, where no clear depletion of Verrucomicrobiae was observed in the ascending colon [34]. Another SHIME experiment also described the distal location of *Akkermansia* spp. [184]. In addition, in this model the growth of *Akkermansia*-like spp. was stimulated by black tea and red wine grape extract. Interestingly, in M-SHIME, *A. muciniphila* did not reach high densities as was observed in the distal compartments in the SHIME setup [186]. This might be due to the setup of the M-SHIME model, which is lacking distal colon compartments, where *Akkermansia*-like spp. reached the highest densities [182,184,185]. Recently, a study using the SHIME model demonstrated that *A. muciniphila* is pH- and mucin-dependent [183]. An increase of *A. muciniphila* was observed upon the addition of mucin. When the pH in the distal colon was lowered, a decrease in *A. muciniphila* was observed in comparison to the same compartment with a higher pH. Altogether, these studies suggest that these models can be used to study the effect of environmental parameters and diet on the human gut microbiota in health and disease states.

## 5. Conclusions

To date, the presence of *A. muciniphila* has mainly been associated with the mucus layer of the colon. However, in this review we collected results from other studies and showed that *Akkermansia*-like sequences have also been found to be present in other anatomical regions of the digestive tract and human breast milk. In short, these regions are the oral cavity, pancreas, bile ducts and gallbladder, small intestine, large intestine, and appendix (Figure 1b). The environmental parameters (e.g., pH, oxygen and nutrient availability) differ among anatomic regions of the human body, affecting the growth of *A. muciniphila*. As the aforementioned organs have different functions, the function of *A. muciniphila* might also differ in different regions of the digestive tract. In this review, we proposed hypothetical functions of *A. muciniphila* in these regions; however, further research is needed to confirm its role among the different regions of the digestive tract. Altogether, the presence of *Akkermansia*-like spp. along the digestive tract indicates that this bacterium might have more functions than those known so far. Still, as can be concluded from abundance of *Akkermansia*-like spp., its optimal ecological niche remains the mucus layer in the colon.

## Supplementary Materials

The following are available online at http://www.mdpi.com/2076-2607/6/3/75/s1: Figure S1: Detailed maximum likelihood phylogenetic tree (RAxML) including *Akkermansia*-like sequences derived from large intestine and ileum biopsies and Chinese *A. muciniphila* strains; Figure S2: Detailed Neighbor Joining phylogenetic tree including *Akkermansia*-like sequences derived from large intestine and ileum biopsies and Chinese *A. muciniphila* strains; Figure S3: Detailed maximum likelihood phylogenetic tree (RAxML) including *Akkermansia*-like sequences derived from fecal samples, large intestine, and ileum biopsies and Chinese *A. muciniphila* strains.
