# Supplementary material for: Akkermansia muciniphila in the Human Gastrointestinal Tract: When, Where, and How?
Source: Microorganisms. 2018 Jul 23;6(3):75. doi: 10.3390/microorganisms6030075 (PMC6163243; doi:10.3390/microorganisms6030075)
Supplement: Supplementary file 1 [file microorganisms-06-00075-s001.zip › Figure S3.pdf]

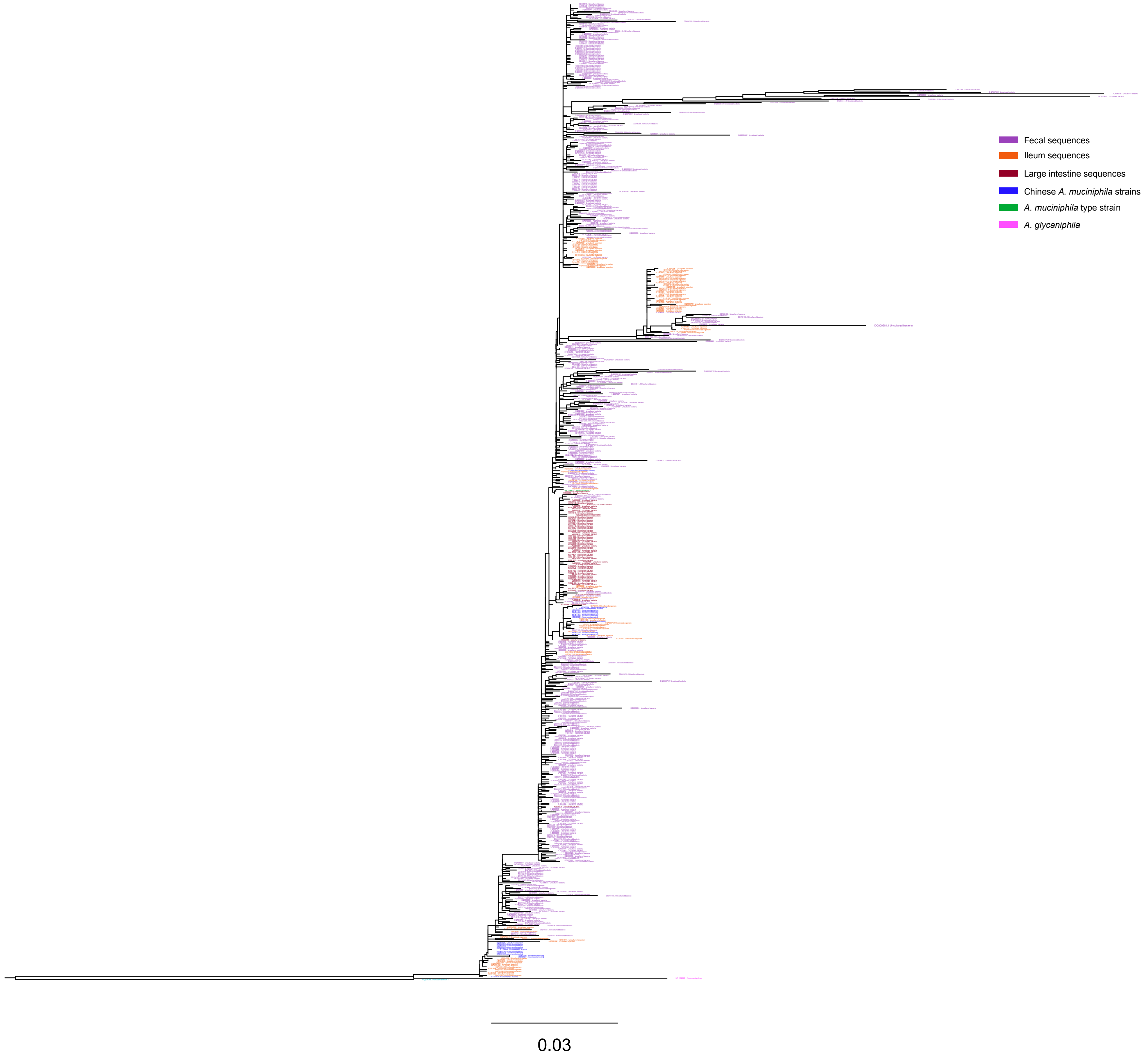
Fecal sequences
Ileum sequences
Large intestine sequences
Chinese *A. muciniphila* strains
A. muciniphila type strain
A. glycaniphila
DQ806281.1 Uncultured bacteriu
0.03
