# Supplementary figures and images for: Akkermansia muciniphila in the Human Gastrointestinal Tract: When, Where, and How?
Source: Microorganisms. 2018 Jul 23;6(3):75. doi: 10.3390/microorganisms6030075 (PMC6163243; doi:10.3390/microorganisms6030075)

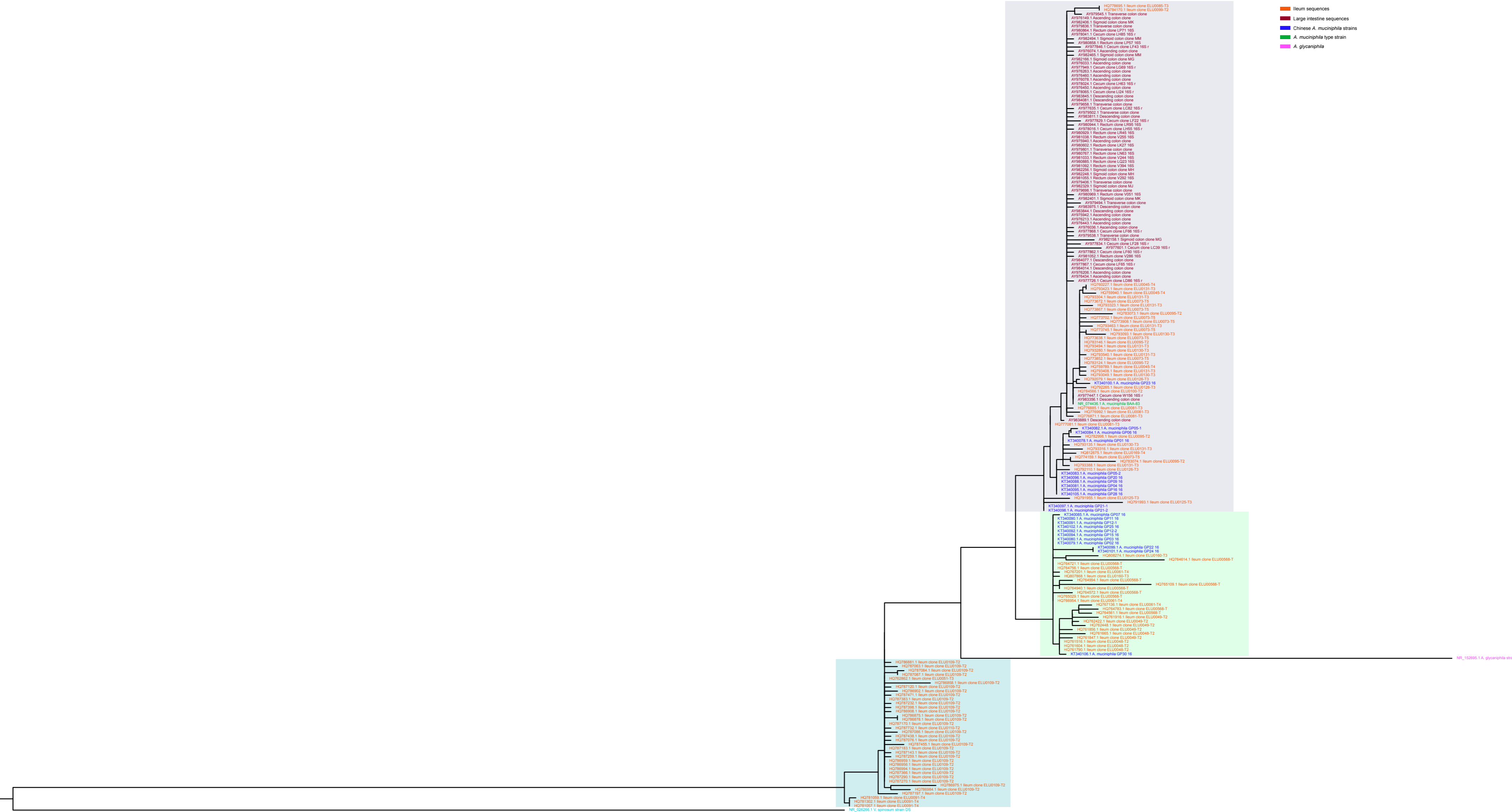

Supplement: Supplementary file 1 [file microorganisms-06-00075-s001.zip › Figure S1.pdf]

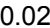

Supplement: Supplementary file 1 [file microorganisms-06-00075-s001.zip › Figure S2.pdf]
